# Simulating cable corridors based on terrestrial LiDAR data

**DOI:** 10.1007/s10342-024-01673-1

**Published:** 2024-03-26

**Authors:** Carl O. Retzlaff, Christoph Gollob, Arne Nothdurft, Karl Stampfer, Andreas Holzinger

**Affiliations:** 1 Human-Centered AI Lab, Department of Forest and Soil Sciences, Institute of Forest Engineering, University of Natural Resources and Life Sciences, Vienna, Peter-Jordan-Strasse 82, 1190 Vienna, Austria; 2 Department of Forest and Soil Sciences, Institute of Forest Growth, University of Natural Resources and Life Sciences, Vienna, Peter-Jordan-Strasse 82, 1190 Vienna, Austria

**Keywords:** Optimization, Cable yarding, Mechanical simulation, Digital twin

## Abstract

This article introduces a new basis for optimising cable corridor layouts in timber extraction on steep terrain by using a digital twin of a forest. Traditional approaches for generating cable corridor layouts rely on less accurate contour maps, which can lead to layouts which rely on infeasible supports, undermining confidence in the generated layouts. We present a detailed simulational approach which uses high-resolution tree maps and digital terrain models to compute realistic representations of all possible cable corridors in a given terrain. We applied established methods in forestry to compute feasible cable corridors in a designated area, including rope deflection, determining sufficient tree anchors and placing intermediate supports where necessary. The proposed individual cable corridor trajectories form the foundation for an optimised overall layout that enables a reduction of installation and operation costs and promotes sustainable timber extraction practices on steep terrain. As a next step we aim to mathematically optimise the layout of feasible cable corridors based on multiple criteria (cost, ergonomic aspects, ecological aspects), and integrate the results into an user-friendly workflow.

## Introduction

Cable yarding continues to be an efficient and effective harvesting system for the extraction of timber on steep terrain. Modern European silvicultural strategies, together with environmental pressures, result in smaller harvest areas. This has lowered extraction volumes and led to a shift from clear-cut to single tree extraction. Yarder installation time has, especially as a proportion of the extraction time, increased significantly, resulting in higher total extraction costs (Schweier et al. 2020). Digital twins of the forest allow optimizing the cable corridor layout in a way that minimizes installation and operation costs while improving forest risk management (Buonocore et al. 2022). Digital twins also lay the groundwork for enabling methods of artificial intelligence (AI) in forestry, promising further advances in the reduction of costs and environmental impact (Holzinger et al. 2022).

Traditional routing methods were first based on manual, then on automated planning using geographic information system (GIS) maps generated by remote sensing approaches. The low resolution of the data often used can lead to inaccurate representations of the forest. As a result, these methods may compute mathematically optimal cable road layouts that depend on unfeasible support trees and anchor. Bont et al. (2019) for example use a tree volume estimation provided by forestry authorities with 10$\times$10 m and 15$\times$10 m resolution respectively, while Stückelberger et al. (2007) based their optimization of forest road networks on a 10$\times$10 m resolution grid as well.

A general issue with data acquired from aerial or above-canopy sources, be it LiDAR or imagery, is that it is challenging for the light to penetrate through trees. This means that precise floor-level positions of the trees or their trunks, as well as trunk diameters, are unavailable. While it is possible to derive this information regressively from the position and height of the tree tops, such estimates are associated with significant errors, rendering them inadequate for applications requiring accurate representations of trees and underlying terrain (Carreiras et al. 2006). Therefore, we relied on terrestrial PLS (personal laser scanning) to obtain highly accurate positions of individual trees, information on the trunk structure, trunk diameter variations, tree height, and consequently, the volume of each individual tree, as well as high-resolution digital elevation models.

This study presents an approach to compute various feasible cable corridors in a designated logging area, utilizing high-resolution DTMs and tree maps. This forms the basis for an optimization process which computes the most cost-efficient combination of intermediate supports and anchor points, minimizing the environmental impact and maximizing worker safety at the same time. We aim to answer the following research question with this paper: is it feasible to compute a set of realistic cable corridors based on detailed tree maps and DTMs to enable the automatic generation of optimal cable corridor layouts?

## Related work

### LiDAR-based modelling

Light Detection and Ranging (LiDAR) technology has been widely used in forestry to generate high-resolution data of forest environments (Newnham et al. 2015). LiDAR works by emitting laser pulses, which then bounce back from the material they encounter to the sensor and are recorded to create a 3D point cloud of the area (Buchelt et al. 2024; Ehrlich-Sommer et al. 2024). Personal laser scanning (PLS) is a common approach to LiDAR scanning, where a person (as opposed to other vehicles such as drones or remote-controlled cars) carries the LiDAR sensor and conducts the scanning process. In a forestry context, LiDAR technology can provide precise information on the forest terrain, tree location, height, and canopy structure (Dubayah and Drake 2000), with several studies aiming to improve the accuracy and robustness of estimating those parameters from the generated point clouds (Witzmann et al. 2022).

It is important to distinguish between terrestrial LiDAR and aerial LiDAR, both of which have unique applications in the field of forestry. Terrestrial LiDAR involves using ground-based laser scanning systems to capture detailed 3D information of trees and their surroundings. This technology provides highly accurate measurements of tree positions, trunk structure, diameter, and volume, making it ideal for precise forest inventory monitoring. On the other hand, aerial LiDAR utilizes airborne platforms to collect data from above the canopy, enabling broader coverage of large forest areas.

As mentioned previously, LiDAR offers the advantage of extensive spatial coverage, but struggles to penetrate dense tree canopies. Terrestrial LiDAR excels in capturing precise tree-level data, but has limitations in terms of coverage and is often more expensive to obtain due to the manual labour involved (Popescu and Wynne 2004). While LiDAR-based simulation has the potential to facilitate different planning and evaluation processes (Lato et al. 2009), it is important to note that the accuracy and quality of the data obtained depends on several factors, such as the instrument used, the density of the point clouds, and the weather conditions (Hancock et al. 2014).

Aerial LiDAR systems are generally not suitable for obtaining precise and comprehensive data regarding individual trees, due to the sparse representation of tree stems in their three-dimensional point clouds and the significant influence of the quality and quantity of field reference data on the derived information (Liang et al. 2014). We therefore aim to show how applying terrestrial laser scanning and the subsequent high-resolution data can be beneficial to applications in forestry.

### Layout optimization

In the past, optimal cable corridor layouts and intermediate support locations on ground profiles were determined through intuition or trial and error. Pestal (1961) proposed rules of thumb for estimating cable deflection in 1961 that are still used today. This approach involves drawing the shape of an unloaded skyline over the ground profile, placing intermediate supports at protruding profile points, and evaluating cable span clearance. Sessions (1992) introduced an automatic search for alternative procedures, while Chung and Sessions (2003) used a heuristic algorithm to eliminate intermediate supports if the ground clearance exceeded the minimum required. While most of these approaches produced feasible solutions, they did not guarantee optimality.

A similar problem to determining optimal cable corridor layouts lies with the location allocation problem, which aims to minimize the overall cost of distributing facilities across a given area to ensure optimal coverage. A common example for this problem is as a supermarket trying to find the optimal location for a new store, where the location minimizes the distance to as many customers as possible. The location allocation problem has been extensively researched since the 1960s, leading to various problem formulations such as the p-median, simple plant location, and location allocation models (Bont and Church 2018). Dykstra and Riggs (1977) introduced optimization methods to identify cost-minimal logging units and allocate equipment to them, which falls under the “facility location” problem class and is a “cascading fixed charge” structure. For small problems, 0–1 integer programming systems can provide mathematical optimal solutions, but approximation algorithms are needed for larger practical problems. This approach was limited to clear-cutting areas with a radial cable corridor pattern and fixed cable corridor length. Later studies attempted to optimize concurrent layouts for both the road network and harvesting, such as the methodology of Chung et al. (2004) for a cable-logging operation and network plan based on multiple optimization approaches, and a cost-optimal road network and technology assignment for harvesting units (Epstein et al. 2006). While these approaches have made progress, their reliance on heuristic algorithms limits the ability to identify the gap between achieved and optimal solutions.

In more recent publications, Bont and Heinimann (2012) developed an approach that uses nonlinear cable mechanical assumptions to design an optimal intermediate support layout, focusing on a more detailed modelling approach. In a subsequent publication, Bont and Church (2018) also introduced a set-covering model (SCM) and a bounded set-covering model (BSCM) for solving cable corridor layout problems, applying the modelling technique at a large scale. Bont et al. (2019) presented a multi-objective approach that balances the need to reduce environmental impact with the demand for cost-effective harvesting, with the goal of facilitating the optimization process. By means of more computing power and better mathematical models, the goal of determining optimal layouts had been reached over the years.

One major limitation of these approaches is the reliance on low-resolution aerial GIS data, resulting in possibly infeasible layouts. This can be highly detrimental to the adoption of the generated results, because users will quickly lose trust in the generated layouts if multiple cable corridors are not working because of the lack of sufficient anchoring or other obstructions not covered by the underlying maps. To overcome this limitation, we propose incorporating high-precision forest stand maps generated by personal laser scanning and simulating cable corridors based on this information. This requires utilizing given techniques for computing individual cable corridors and adopting them to automatically and rapidly compute the feasibility of multiple cable corridors. The resulting cable corridor layouts are highly realistic, cost-efficient, and minimize environmental impact while maximizing working safety. By doing this, we greatly improve the reliability of the generated cable corridor layouts and with that aim to increase their adoption in forestry. This adoption can help to accelerate the move towards smaller harvest areas, lower extraction volumes, and the shift from clear-cut to single tree extraction.

## Methods

The aim of this work is to start bridging the gap between realistic one-off cable corridor planning based on individual trajectories as seen in Bont et al. (2022) and area-wide, but less reliable layout optimization based on GIS data such as Bont (2012). We leveraged the PLS-based tree maps and digital terrain models (DTM) to automatically generate possible cable corridors by implementing different approaches for computing cable corridor properties. The corresponding code can be found and replicated on the GitHub repository Retzlaff (2023a) of this project.

The data acquisition and processing was not part of this study, but is described in detail since it is are essential for this work. For further information, refer to Gollob et al. (2020, 2021, 2024).

The study area was a steep terrain in a forested region, situated near the village of Mautern (47^∘^24’3.68” N 14^∘^49’34.42” E) in the federal state of Styria, Austria. The forest stand encompasses an area of 1.62 hectares with an average slope of 62.6%. The stand is predominantly comprised of Picea abies with a small proportion of Pinus sylvatica. The forest stand contains 480 stems, equating to a density of 296 stems per hectare, with a minimum diameter at breast height (DBH) threshold of seven centimeters. On average, the trees have a DBH of 35.6 cm and a height of 25.6 m. Utilizing individual tree data and taper curve estimation routines, a total volume of 811.5 cubic meters (500.9 cubic meters per hectare) was estimated.

High-resolution terrain and tree maps were collected through personal laser scanning (PLS) to obtain accurate data for cable corridor layout optimization. The 1.62 ha forest stand was scanned using a GeoSLAM ZEB Horizon personal laser scanner (Geoslam Ltd 2023). The stand was scanned in loops 10–20 m apart in one scan pass. The maximum scanning range of the system is 100 m, and the acquisition rate is 300,000 points per second. After data collection in the field, 3D point clouds were generated using a SLAM (Simultaneous Localization And Mapping) algorithm (Gollob et al. 2020). Terrain, individual tree, and stand parameters were extracted using automatic routines developed at the Institute for Forest Growth of the University of Natural Resources and Life Sciences with the R programming language (Tockner et al. 2022; R Core Team 2023).

The following parameters were calculated from the point cloud: XYZ center coordinates of the tree, tree height, tree volume, diameter at breast height (DBH), taper curves for estimation of diameters along the stem, and a digital terrain model (DTM) with 1 m resolution. For more information on laser scanning and calculation of terrain and tree parameters, refer to Gollob et al. (2020), Tockner et al. (2022). These parameters then form the basis for optimizing the cable corridors.

The simulation of cable corridor feasibility and parameters is based on multiple computational methods established in the forestry domain to evaluate cable corridors. If not specified otherwise, equations and their definition are based on Bont (2012), which provides a comprehensive overview of methods for rope deflection computation and their required parameters. Multiple factors concerning details about anchoring and rope tensions processes are derived from a discussion with an expert in forest technology (Gollob 2022), while the geometric approaches for determining loads on the cable corridor itself and supports are based on Stampfer (2000).

Initially, Mr. Gollob participated in an interview as a forest technology expert, assessing the feasibility of our proposed cable corridor computation approach and reviewing preliminary results. He has authored several publications related to LiDAR scanning and the creation of high-resolution tree maps, demonstrating his expertise in generating digital twins for forestry. Additionally, Mr. Gollob owns a professionally managed forestry area and a cable yarding system, with over 15 years of practical experience in its use. Following the interview about the evaluation of our cable corridor computations, he was consulted several times about various details of the cable corridor planning process. As a result of his continued involvement and insights, he joined the publication as co-author and contributed to the final publication with his expert knowledge on forestry topics.

Table 1 provides an overview of different assumptions made for computing a given cable corridor, with factors such as wood E-modulus, cable weight, etc.

**Table 1:** Overview of assumptions for different variables

Factor name	Default value	Explanation	Source
*Support tree*			
Safety factor	3	The safety factor for Eq. 4	Gollob (2022)
Wood E-modulus	8 kN/mm^2^	The average E-modulus of wood	AmewsWeb (2023)
Attachment height of support anchor	2 m	Additional height to the cable corridor where the supports are attached on the support tree, influencing the buckling force	Gollob (2022)
*Tower anchors*			
Outer angle range	20^∘^ to 5^∘^	The allowed angle from the outer anchor with regard to the central anchor	Gollob (2022)
Maximum center tree to slope angle	3^∘^	The maximum allowed angle from the tower to anchor tree cable corridor to the hill slope	Gollob (2022)
Anchor to tower distance range	15 to 40 m	The allowed distance from the tower to the anchor trees	Gollob (2022)
*Cable corridor*			
Maximum slope deviation	45^∘^	The maximum deviation of the cable corridor from the hill slope. Cable corridors with a deviation greater than this value will not be considered.	Bont et al. (2019)
Tower height	11 m	Height of the tower support	Bont et al. (2022)
Minimum height of rope	3 m	Minimum clearance between ground and rope	Bont et al. (2022)
Rope weight	1.46 kg/m	Rope weight in kilograms per meter, assuming an 18 mm steel rope	Teufelberger (1999)
Pull rope weight	1 kg/m	Pull rope weight in kilograms per meter	Teufelberger (1999)
Applied load	10 kN	Vertical load applied to the cable corridor	Bont et al. (2022)

Rope deflection computation is conducted as per Pestal (1961). This method, also referred to as the “Pestal approach”, calculates the amount of deflection in the rope between two supports by taking into account the sag due to the weight of the rope and any additional loads on the rope. Refer to the publication of Bont and Heinimann (2012) for a detailed description of the approach and the required parameters. The amount of deflection in the rope affects the tension and the curvature of the rope, and is determined by the following terms:

- *y*: width of the whole section
- *b*: length of the whole section
- $H_t$: horizontal force component of the cable corridor
- *q*: vertical force
- *c*: rope length
- $$q_s$$: rope weight
- $\overline{T}$: tensile force at the center span
- $T_0$: basic tensile force
- *h*: height difference between two supports
- *y*: deflection at point *x*
- *z*: height of the center span

We computed both the tensile force at the center span 1 and the horizontal tension in the cable corridor 2 to then yield the load path 3:

1 $$\begin{aligned} \overline{T}= & {} T_0 + q_s * (h/2+z) \end{aligned}$$

2 $$\begin{aligned} H_t= & {} \overline{T} * b/c \end{aligned}$$

3 $$\begin{aligned} y= & {} \frac{x*(b - x)}{H_t * b} * Q +\frac{c*q}{2} \end{aligned}$$

The maximum force that a support can withstand is then computed according to Pestal. This method calculates the maximum force in newton that a support can bear based on the tension and curvature of the rope at that point (Pestal 1961). It ensures that the supports are sufficiently strong to withstand the forces exerted by the cable carriage system. *M* is the maximum pressure the support can withstand in newton (N), *d* the diameter of the trunk in cm, *E* the e-Modulus of the wood in N/cm^2^, *l* the buckling length where the load is attached in cm, and *S* the safety factor.

4 $$\begin{aligned} M = \frac{\pi ^ 2 * E * \pi * d^4 }{l^2 * 64* S} \end{aligned}$$

Equation 5 finally defines the holding force a tree can provide as anchor in newton, where *DBH* is the diameter at breast height in decimeter and *S* the safety factor. The reduction factor is normally determined on a per-tree basis and reflects circumstances like tree vitality and root formation, floor conditions etc. Since these are not reflected in the data available, we set the safety factor to a conservative average (5).

5 $$\begin{aligned} M =\frac{DBH^2 * 10.000}{S} \end{aligned}$$

**Fig. 1:**
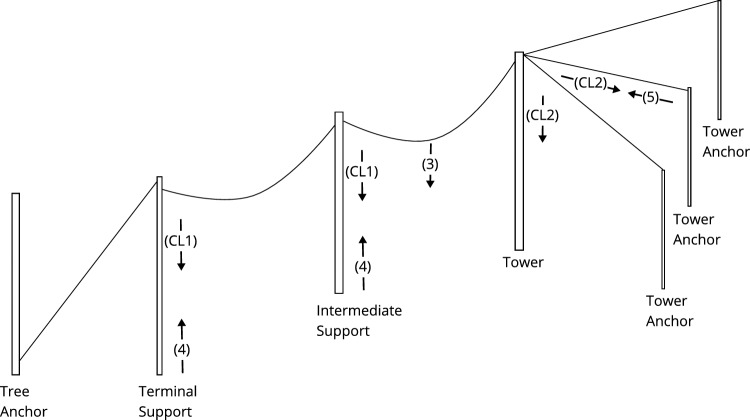
Overview of terms and equations applied for generating individual cable corridors. Numbers in parentheses correspond to the given equations in Sect. 3, CL1 corresponds to the code computing forces on the anchor, and CL2 to the code computing the tower forces

Figure 1 provides a comprehensive overview of which equation is applied to which aspect of the cable corridor computation. Note that all equations mentioned in this section are directly based on the literature, whereas the other equations and approaches developed within this paper are described in the results section.

To validate the accuracy of the simulated results, the generated layout was first evaluated by forestry experts with regard to its feasibility, taking into account the terrain, slope and layout of trees (Gollob 2022). In addition to consulting with the forestry expert, the results produced by our process were also compared against SEILAPLAN layouts (Bont et al. 2022).

## Results

All underlying code and the results of the planning process are publicly available in the GitHub repository Retzlaff (2023a).

### Data preparation for the digital twin

**Fig. 2:**
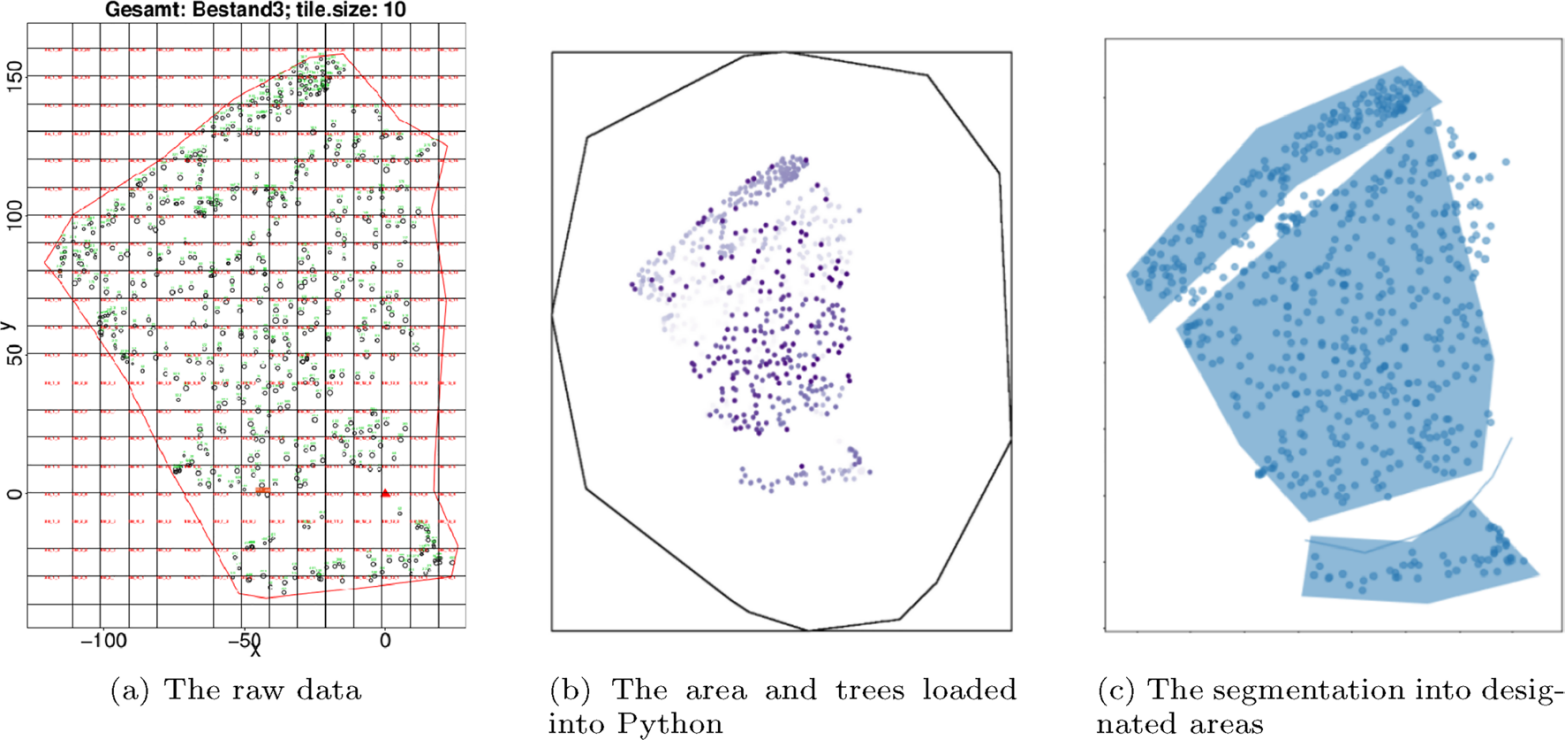
Loading process and segmentation of the raw data into different functional areas

The process of creating the cable corridor layout involves importing the tree map data in Comma-Separated Values (CSV) format and coupling it with the digital terrain model (DTM) to obtain a comprehensive dataset of the forest terrain. The trees are then separated into three categories, namely tower anchors, harvestable trees, and tree anchors, based on their location in relation to the tower anchors and end area of the terrain. See the last Fig. 2 for an example of the designated areas, as well as the road line along which the tower points are placed. The hill slope direction is then determined using the DTM, and a set of possible tower points are established along the proposed road. These tower points are placed at a given interval (by default every 2 m) and enabled us to later generate and test all possible combinations of cable corridors. The results of this process are dependent on the quality and accuracy of the input data, which should therefore be of high resolution and precision.

### Approaches for determining load on supports, tower and anchors

The force on intermediate supports as well as on the anchor cable is determined by our implementation of the mechanical computation (Retzlaff 2023b). We implemented the approach applied by Stampfer (2000), which determines the force on the support by evaluating the distance between loaded and empty cable corridor after applying the force on the cable. The higher the distance between sloped and empty cable corridor, the higher the force on the support. This approach helped to ensure that the forces are evenly distributed and that no single support or segment is subjected to mechanical overload.

The force on the cable corridor tower is computed similarly, based on the geometric solution proposed by Stampfer (2000). The code translates the geometrical step-by-step instructions to an automatic computation of the exerted force on the tower (Retzlaff 2023b). Refer to Fig. 6 for a comparison of the resulting layout and the geometric process.

### Computing cable corridors

The main function of the cable corridor layout optimization process consists of first computing all possible combinations of tower points and tail anchors. These combinations are then subjected to a filtering process based on a maximum allowable level of slope deviation to ensure that the cable corridor remains safe and stable during installation and operation. The filtered tree combinations are then evaluated based on the availability of an eligible support tree. An eligible support tree is one that is located within a distance of 0.5 to 2 ms from the proposed cable corridor trajectory and supports a buckling force greater than 10 kilonewton (kN).

Possible triplets of anchor trees for each tower point are computed. These triplets are used to determine the optimal placement of the tower and ensure that the cable corridor setup is stable. After generating the anchoring, only lines that contain at least one viable anchoring configuration are selected for further analysis.

**Fig. 3:**
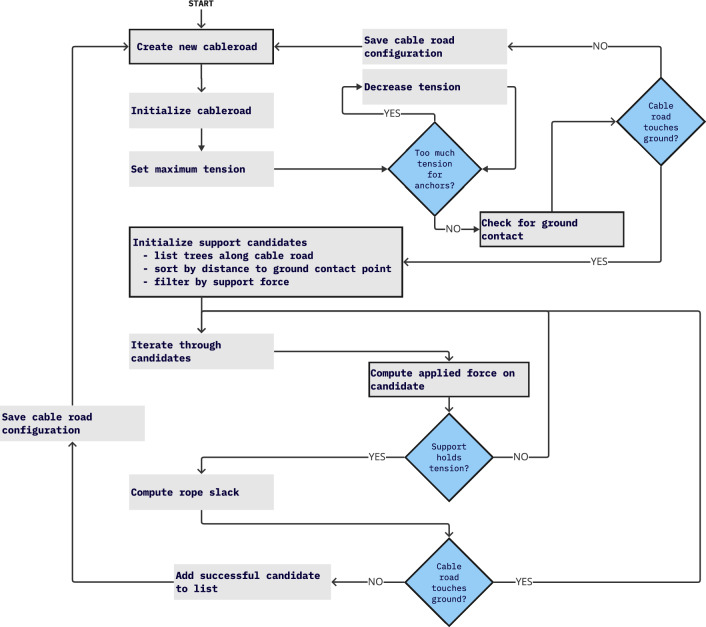
Flow chart of the process for determining the amount and position of required supports for a given cable corridor. Steps where the PLS data was leveraged to receive more accurate results are highlighted with a black border, either by using detailed information on single trees to compute accurate forces or relying on the DTM to test for ground collisions in high resolution

The next step in the optimization process is to compute the required supports for the selected cable corridor trajectories. This involves determining the optimal locations for intermediate supports along the cable corridor layout to ensure that the cable remains stable and minimizes its impact on the environment. A flowchart of the computation process is shown in Fig. 3, which illustrates the steps taken to generate a stable and feasible cable corridor layout.

The tensioning process of the cable corridor is oriented at a top-down approach, i.e. we start with the highest possible tension and lower it until all components of the cable corridor can support it. The tension of the cable corridor is directly set to the maximum force the cable itself supports (minimum breaking force of the cable, 170 kN, divided by 2 as safety factor, resulting in 85 kN). Then, the tension is lowered in a step-wise fashion in decrements of 10 kN until both the tower, anchors and intermediate supports can withstand the occurring forces. The decrements are set at 10 kN to allow acceptable performance, since each decrement requires recomputing both cable slope and cable corridor tensions. The step size can however easily be changed if the user wants a more fine-grained search.

**Fig. 4:**
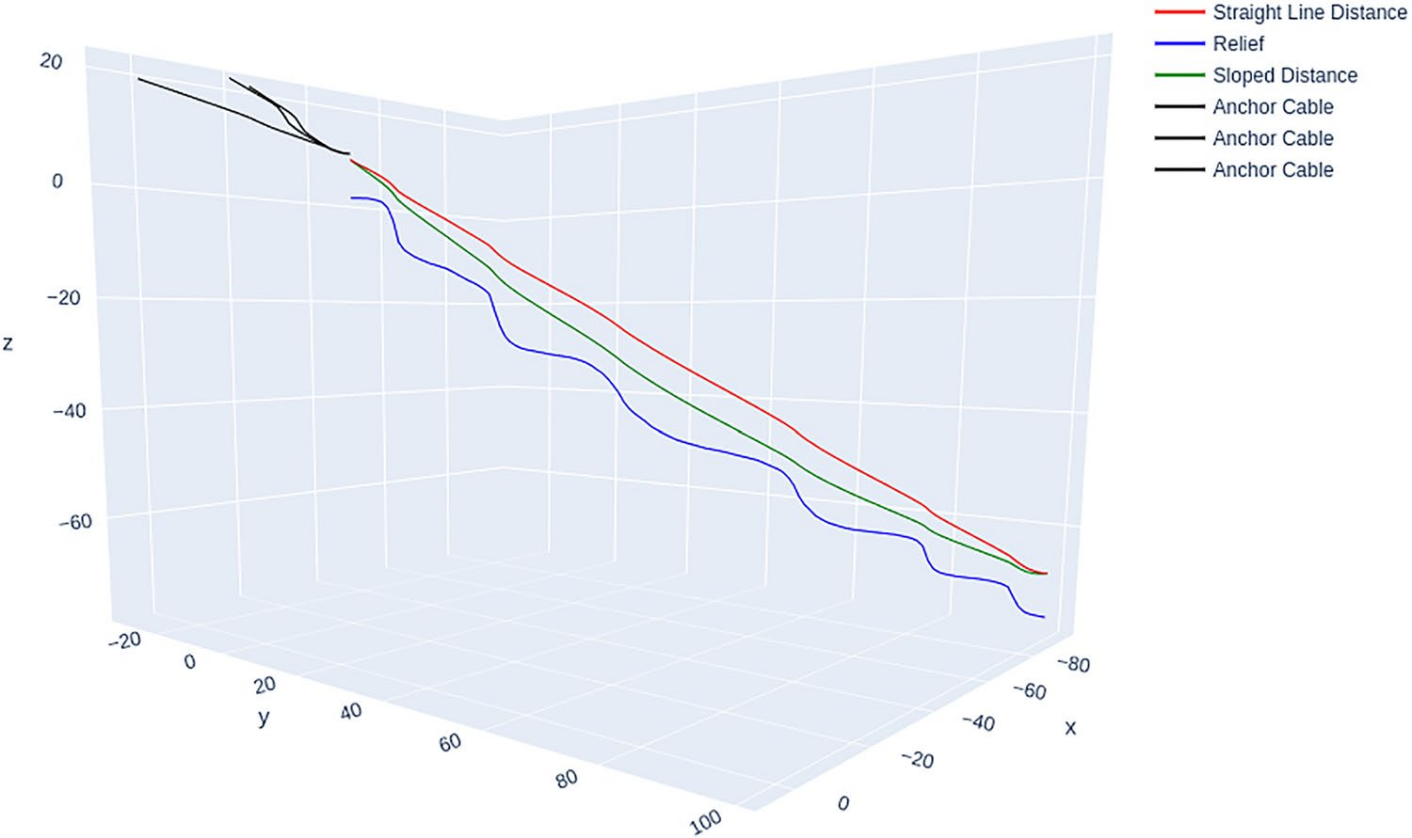
Detail view of a single cable corridor with the direct line, sloped line, terrain topography and anchors with 65 kN rope tension

In the cable corridor layout optimization process, the tensioning process may sometimes fail to produce a cable corridor layout without ground collisions. When this occurs, the optimization algorithm creates additional supports at the points where the cable last touched the ground, and attempts to remove any collisions between the cable and the environment. The process of creating additional supports and removing collisions is repeated until either a stable and feasible cable corridor layout is obtained, or the line is declared unfeasible (if more than four supports are required). By iteratively adjusting the placement and number of supports, the optimization algorithm produces a cable corridor layout that meets the desired criteria (Fig. 4). This process is consistent with the approach (Pestal 1961).

**Fig. 5:**
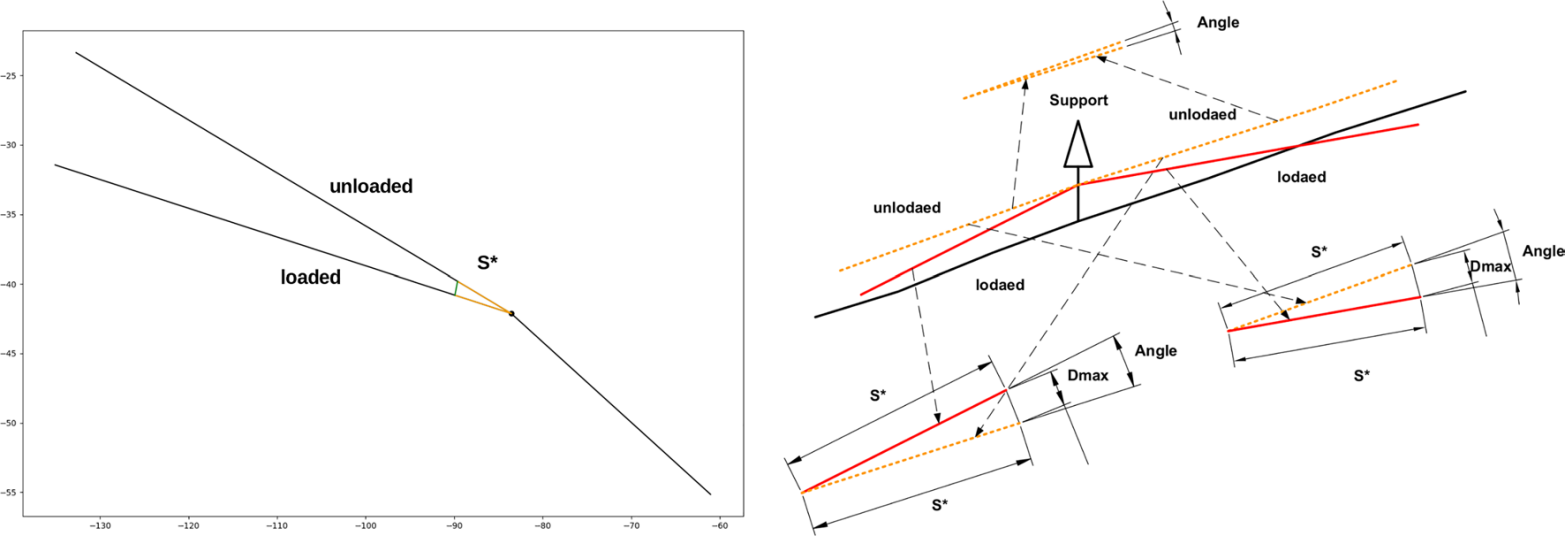
Computation of force on the support with our implementation in comparison to Stampfer (2000)

**Fig. 6:**
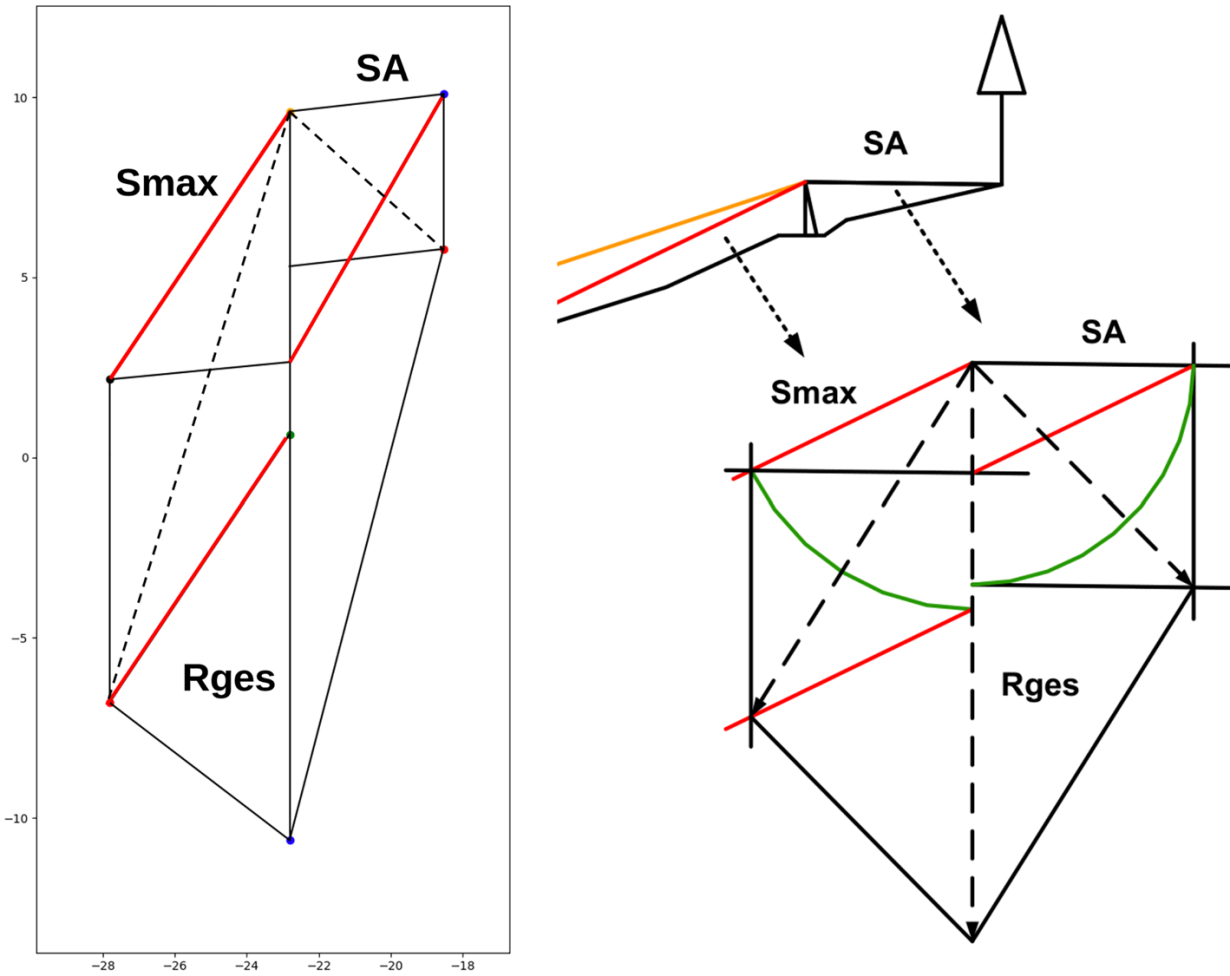
Computation of forces on anchor and tower with our implementation in comparison to Stampfer (2000)

Central components of this process are the determination of forces on tower and anchors as well as the determination of forces on intermediate supports. Both approaches are based on the geometrical computation by Stampfer (2000). Figure 5 shows the construction of angles and forces at the support and compares it against the geometric approach of Stampfer (2000). Figure 6 shows the construction of angles and forces for the tower situation, while Fig. 4 shows an individual resulting cable corridor. All successfully computed lines (see Fig. 9 for an example) are then saved and prepared for the optimization of the layout.

### Evaluation

**Fig. 7:**
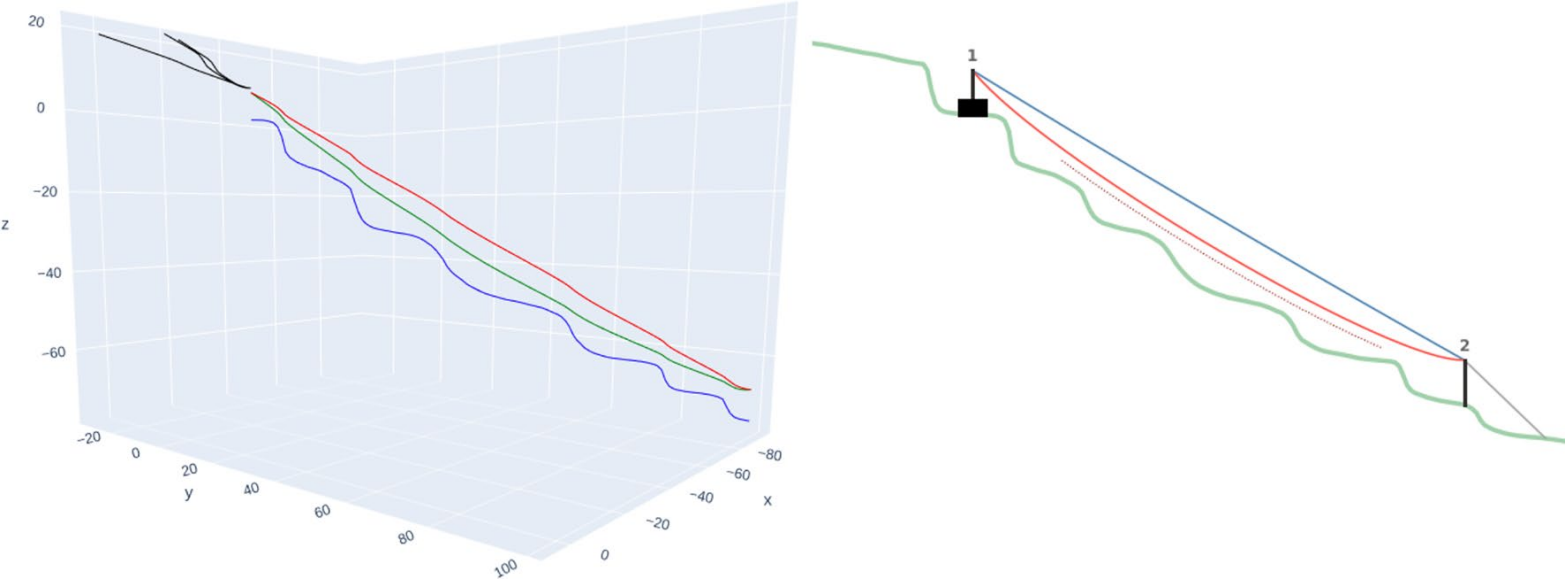
View of a single cable corridor computed by the presented approach compared to the result by SEILAPLAN

The results were evaluated by an expert interview (Gollob 2022), indicating that the simulation adequately captured the behaviour of the cable corridor situation. Specifically, we observed that the tension and curvature of the cable corridor closely matched the simulated results, and the maximum forces exerted on the supports were within the predicted range. Computing all 54 possible cable corridors for an area with 633 trees (22 of those possible anchor trees with a DBH larger than 30 cm and 75 possible cable corridors) requires 12 min 32 s (± 12 s) on a laptop with an Intel Core i7-1270P and 32GB RAM. To evaluate our results, we additionally compared individual cable corridors to cable corridors computed by the SEILAPLAN program. Figure 7 shows the comparison of a cable corridor computed with the presented approach and a similar SEILAPLAN implementation. We see that the rope path is very similar, with the rope deflection being 6.24 m in our implementation and 5.82 m in SEILAPLAN. The algorithm determined an optimal rope tension as 65 kN, which places a load of 47 kN on the anchor and 224 kN on the tower, requiring 931 ms (± 8 ms) for the computation.

**Fig. 8:**
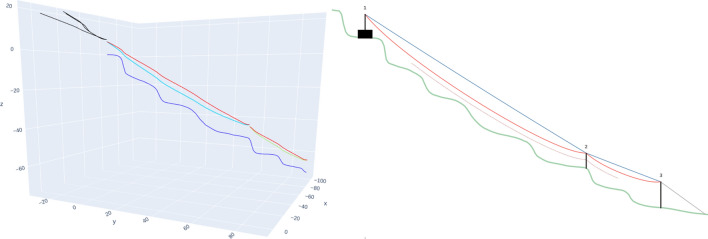
View of a cable corridor with an intermediate support computed by the presented approach compared to the result by SEILAPLAN

**Fig. 9:**
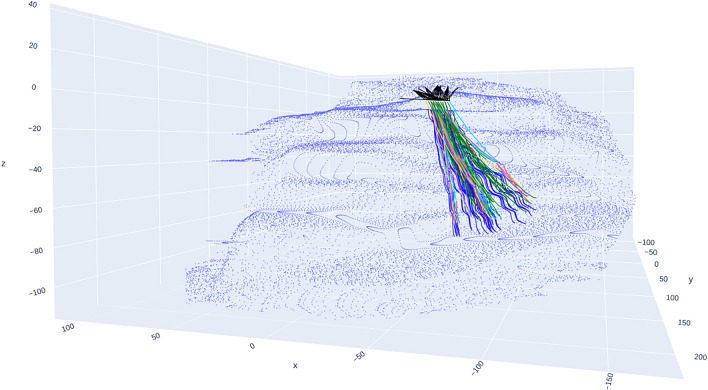
Overview of the computed feasible cable corridors overlaid on the terrain topography

Figure 8 shows the computation of a cable corridor with supports. The results again show a close correspondence of both cable corridor trajectories. Rope deflection is 7.12 and 4.69 ms, compared to 6.54 and 4.28 ms determined by SEILAPLAN. In this configuration, a rope tension of 65 kN is applied, resulting in 10 kN load on the intermediate support, 47 kN load on the anchor and 220 kN load on the tower. The computation for such a configuration took 182 ms (± 33 ms).

Based on the comparison with SEILAPLAN simulations, we concluded that our approach allows to adequately represent the behaviour of the cable carriage system and can be used as a basis for designing and optimizing cable corridor layouts.

## Discussion

In this study, we have introduced a novel approach for generating realistic cable corridor layouts for timber extraction in steep terrain. Our method integrates high-resolution terrain maps and tree maps generated by PLS, aiming to bridge the gap between manual approaches and automatic cable corridor layouts based on GIS data. While we have conducted initial evaluations, involving discussions with forestry experts and comparisons with simulations from the widely used SEILAPLAN plugin, it is important to highlight the need for further testing in real-world scenarios to establish the practicality and feasibility of our proposed method.

The PLS-based DEMs and forestry maps offer highly accurate measurements of tree positions, trunk structure, diameter, and volume, making it well-suited for fine-scale forest inventory and monitoring, whereas traditional satellite imagery struggles to capture detailed information on individual tree attributes. Consequently, traditional optimization approaches, such as those presented by Bont et al. (2019), which rely on satellite-based imaging techniques, cannot generate layouts with comparable reliability and accuracy. We showed how, in contrast, the use of terrestrial LiDAR enables us to obtain accurate and comprehensive data for simulating realistic cable corridors, forming the basis for optimizing the layout of cable corridors in the studied forest stand. With this, a key consideration to bear in mind is the dependence of our approach on accurate tree map data and digital elevation models. Acquiring such data requires specialized equipment and trained personnel for data collection and modeling. Consequently, the availability and quality of these inputs may vary, potentially impacting the reliability and accuracy of the generated cable corridor layouts. Future research should focus on addressing the challenges associated with data collection and processing to enhance the robustness of our approach.

Additionally, we acknowledge that our model does not account for unforeseen environmental or terrain factors, such as damaged trees or changing ground conditions. While we have incorporated a high safety factor in the computation of cable corridor forces to mitigate this uncertainty, it is important to recognize that unknowns and special situations may still limit the applicability of the generated layouts. Future work should explore ways to integrate dynamic factors and adaptability into the cable corridor layout generation process to enhance its resilience and effectiveness in diverse environmental conditions.

Looking ahead, the automatic generation of cable corridor layouts holds significant potential for improving timber extraction practices and facilitating its adoption in the field. To maximize the benefits of this approach, we recommend the development of a user-friendly tool suite that enables interactive planning and layout optimization, while also providing explanations for the decisions made by the underlying algorithms. Adopting a human-in-the-loop approach can enhance user interaction and foster more efficient and cost-effective timber extraction practices (Retzlaff et al. 2024).

From a broader perspective, our proposed approach has the potential to promote sustainable timber extraction practices. A mathematical optimization of cable corridor layouts could allow reducing installation and operation costs and minimizing environmental impact. As our approach forms the basis of such an optimization process, it can contribute to the optimization of the overall wood supply chain and encourage sustainable practices within the forestry industry (Müller et al. 2019). Furthermore, our approach can inform the development of new policies and regulations aimed at promoting sustainable timber extraction in areas characterized by steep terrain. However, it is imperative to acknowledge that further research is necessary to fully explore the potential of our approach and understand its broader impact on the forestry industry.

## Conclusion

We have proposed a step-based heuristic approach that lays the groundwork for the mathematical optimization of cable corridor layouts in steep terrains. This automatic optimization of cable corridors has the potential to both reduce costs and foster sustainable forestry practices by identifying the most efficient combination of cable corridors within a specified area. While the feasibility of our computation has been demonstrated in comparison to established simulation approaches, it needs to be verified in real-world scenarios. We recommend future work to focus on a mathematical optimization of cable corridor layouts as well as developing a user-friendly tool suite for interactive layout planning, which could further enhance the adoption of this method. In summary, our approach represents another step towards digitalization in the field of forestry and holds considerable promise for the future of sustainable timber extraction in steep terrains.

## Figures and Tables

**Fig. 10:**
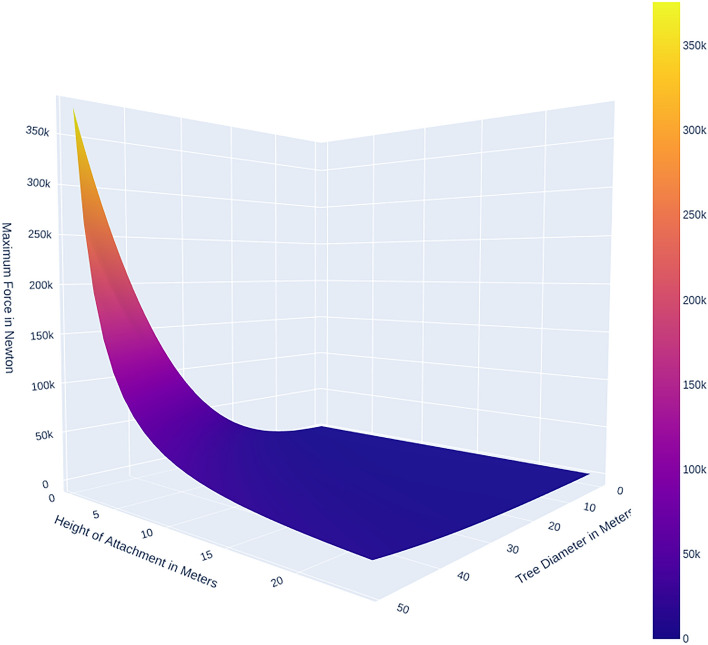
The maximum force allowed by a support based on the height of attachment and the tree diameter

## Appendix: Images

See Figs. 8, 9, and 10.
